# Macronutrient Distribution and Frailty According to Energy Sufficiency

**DOI:** 10.3390/nu18203393

**Published:** 2026-10-16

**Authors:** Yoo-Bin Seo, Jinyoung Shin, Jaekyung Choi

**Affiliations:** Department of Family Medicine, Research Institute of Medical Science, Konkuk University Medical Center, Konkuk University School of Medicine, Seoul 05030, Republic of Korea; 20260144@kuh.ac.kr (Y.-B.S.); jyshin@kuh.ac.kr (J.S.)

**Keywords:** frailty, macronutrients, Acceptable Macronutrient Distribution Range, energy, older adults, KNHANES

## Abstract

Background/Objectives: Nutritional status is a potentially modifiable factor associated with frailty in older adults. However, the association between macronutrient balance and frailty remains understudied and may differ by energy intake sufficiency. We examined the association between adherence to the Acceptable Macronutrient Distribution Ranges (AMDRs) and frailty by energy intake sufficiency. Methods: This cross-sectional study included 2803 adults aged ≥65 years who participated in the 2022 and 2024 Korea National Health and Nutrition Examination Survey. Macronutrient intake was classified using the 2025 Korean AMDRs for carbohydrate (50–65%), protein (10–20%), and fat (15–30% of energy). AMDR adherence required all three ranges to be met. Energy sufficiency was defined as ≥30 kcal/kg/day, and frailty was assessed using a modified Fried phenotype. Survey-weighted logistic regression and restricted cubic spline (RCS) analyses accounted for complex sampling design. Results: Among 925 energy-sufficient and 1878 energy-insufficient participants, frailty prevalence was 6.1% and 9.5%, respectively. AMDR non-adherence was associated with higher odds of frailty in energy-sufficient participants (OR, 5.58; 95% CI, 1.59–19.67) but not in energy-insufficient participants (OR, 1.08; 95% CI, 0.69–1.68). Among energy-sufficient participants, non-adherence to protein (OR, 2.63; 95% CI, 1.02–6.84) and fat (OR, 2.38; 95% CI, 1.19–4.77) AMDRs was significantly associated with frailty; carbohydrate non-adherence was borderline significant (OR, 2.15; 95% CI, 1.00–4.65). RCS analyses showed significant non-linear associations of carbohydrate and protein intake with frailty in energy-sufficient participants. Conclusions: AMDR non-adherence was associated with frailty primarily among older adults with sufficient energy intake. These findings suggest that energy adequacy and macronutrient distribution may represent complementary dimensions of nutritional status in relation to frailty.

## 1. Introduction

Frailty is a geriatric syndrome characterized by reduced physiological reserve and increased vulnerability to stressors, distinct from the normal aging process [1]. It is associated with adverse outcomes, including falls, hospitalization, disability, and mortality [2,3]. Because frailty is potentially preventable or reversible rather than an inevitable consequence of aging, identifying modifiable factors is critical for healthy aging [4]. The development of frailty is multifactorial and is influenced by sociodemographic, behavioral, medical, and nutritional factors [5]. Among these, nutrition is an important, potentially modifiable determinant because inadequate dietary intake may contribute to loss of muscle mass and strength, impaired physical function, and reduced resilience to physiological stress [6]. Older adults may be particularly vulnerable to nutritional inadequacy because age-related changes in appetite, physical function, comorbidities, and social circumstances can adversely affect food intake [7]. Accordingly, nutritional assessment and intervention have become important components of strategies aimed at preventing or delaying frailty [2].

Previous studies examining the relationship between nutrition and frailty have focused largely on protein intake, given its important role in maintaining skeletal muscle mass and function in older adults. However, findings have been inconsistent, with associations varying according to how protein intake was expressed and analyzed, including absolute intake, body-weight–adjusted intake, and the proportion of total energy intake [8,9,10]. In contrast to this substantial body of protein-focused research, comparatively few studies have examined the overall balance of macronutrient intake in relation to frailty. In Korean older adults, Yang et al. (2023) reported that a high proportion of energy from carbohydrate was associated with frailty, and that replacing fat energy with carbohydrate was associated with a graded increase in frailty risk [11]. These findings suggest that the relative balance among macronutrients, beyond the absolute intake of any single nutrient alone, may represent an important yet insufficiently studied factor in the nutritional context of frailty.

The Acceptable Macronutrient Distribution Range (AMDR) provides a standardized framework for evaluating macronutrient balance by expressing carbohydrate, protein, and fat intake as proportions of total energy intake. The 2025 Dietary Reference Intakes for Koreans revised the AMDRs based on accumulating evidence linking macronutrient distribution with long-term health outcomes, lowering the lower boundary for carbohydrate from 55% to 50% of energy and raising that for protein from 7% to 10% of energy [12]. Using the previous Korean AMDR criteria, Kim et al. (2025) found that carbohydrate intake above the AMDR and fat intake below the AMDR were more prevalent among frail older adults, suggesting that deviations from recommended macronutrient distributions may be relevant to frailty [13].

The relevance of macronutrient intake may also depend on whether overall energy needs are adequately met. Adequate energy intake is important for maintaining metabolic and muscle function, and insufficient energy availability may adversely affect muscle protein turnover even when amino acid availability is adequate [14]. In line with this concept, Kang et al. (2025) reported that the association between protein intake and frailty differed according to energy sufficiency and protein source, with inverse associations for total and animal protein observed only among those with sufficient energy intake, whereas plant protein was inversely associated with frailty regardless of energy status [15]. Taken together, these findings suggest that both adequate energy intake and balanced macronutrient distribution may be relevant to frailty, and that the association between macronutrient balance and frailty may differ according to whether overall energy needs are met. We therefore hypothesized a priori that energy intake sufficiency would modify the association between adherence to the AMDRs and frailty. Using nationally representative data from Korean adults aged ≥65 years, we examined the association between adherence to the revised 2025 Korean AMDRs and frailty according to energy intake sufficiency. We further evaluated associations with individual macronutrient distributions and their continuous dose–response relationships with frailty.

## 2. Materials and Methods

### 2.1. Study Design and Participants

**Figure 1:**
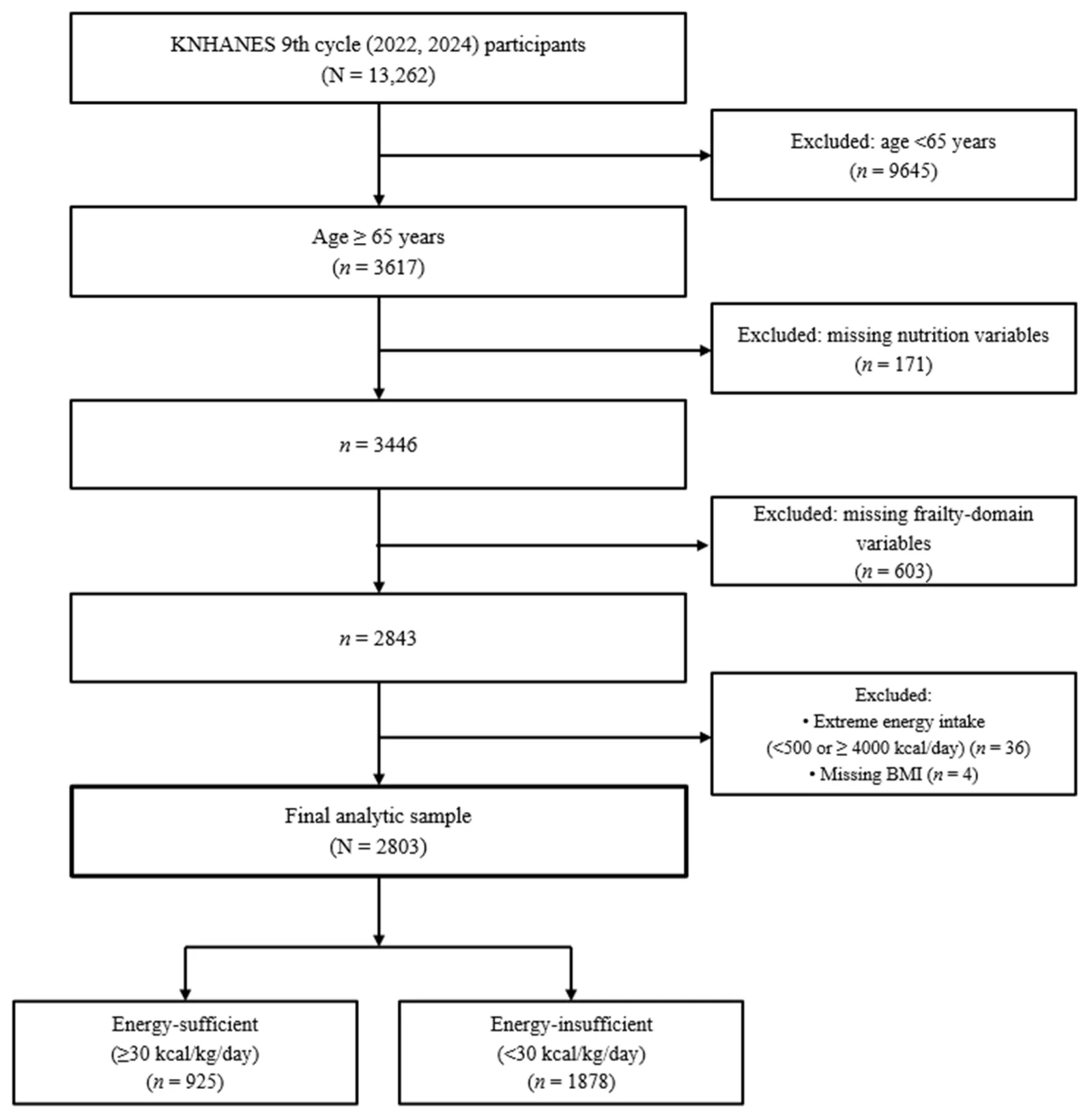
Flowchart of participant selection. Abbreviations: BMI, body mass index; KNHANES, Korea National Health and Nutrition Examination Survey.

This cross-sectional study used data from the Korea National Health and Nutrition Examination Survey (KNHANES) conducted by the Korea Disease Control and Prevention Agency (KDCA). KNHANES is a nationwide, population-based survey designed to assess the health and nutritional status of the Korean population using a stratified, multistage probability sampling design. The present study used data from the 2022 and 2024 surveys, in which the variables required to construct the modified Fried frailty phenotype were available [3]. KNHANES received approval from the KDCA Institutional Review Board for each survey year, and written informed consent was obtained from all participants (approval numbers 2018-01-03-4C-A for 2022 and 2022-11-16-R-03 for 2024). The study population consisted of adults aged 65 years or older. Of 13,262 total participants in the KNHANES 2022 and 2024 surveys, 3617 were aged ≥65 years. After excluding participants with missing dietary-intake data (*n* = 171), missing data on any Fried frailty-phenotype component (*n* = 603), implausible total energy intake (total energy intake < 500 kcal/day or ≥4000 kcal/day; *n* = 36), and missing body mass index (*n* = 4), a final analytic sample of 2803 participants was included (Figure 1).

### 2.2. Assessment of Dietary Intake, AMDR Adherence, and Energy Intake Sufficiency

Dietary intake was assessed using 24-h dietary recall data from KNHANES, including total energy intake and daily intakes of carbohydrate, protein, and fat. The percentage of total energy from each macronutrient (%E) was calculated using conversion factors of 4 kcal/g for carbohydrate and protein and 9 kcal/g for fat. Macronutrient intake was classified according to the AMDRs specified in the Korean Dietary Reference Intakes: 50–65%E for carbohydrate, 15–30%E for fat, and 10–20%E for protein [12]. Participants whose intake of all three macronutrients fell within the respective AMDRs were classified as AMDR-adherent; those with at least one macronutrient outside its range were classified as non-adherent. Daily energy intake was standardized to body weight (kcal/kg/day). Based on the European Society for Clinical Nutrition and Metabolism (ESPEN) practical guideline for clinical nutrition and hydration in geriatrics [16], which recommends 30 kcal/kg body weight/day as a guiding value for older adults, participants were classified as energy-sufficient (≥30 kcal/kg/day) or energy-insufficient (<30 kcal/kg/day). Because the ESPEN recommendation is intended as a practical guiding value rather than a validated cutoff for classifying individuals, the 30 kcal/kg/day threshold was selected a priori as an operational criterion for stratification rather than as a diagnostic cutoff for individual energy sufficiency.

### 2.3. Assessment of Frailty

Frailty was assessed using a modified Fried frailty phenotype adapted to the variables available in KNHANES [3]. The phenotype comprised five components—unintentional weight loss, exhaustion, low physical activity, weakness, and slowness—each assigned one point, with participants meeting three or more criteria classified as frail. The original criteria, the definitions used in the present study, and the rationale for each modification are summarized in Table A1. Unintentional weight loss was defined as a self-reported decrease in body weight of ≥3 kg over the preceding year (response categories: 3–6 kg, 6–10 kg, ≥10 kg) among participants who had not intentionally attempted to lose weight. Exhaustion was defined as having experienced tiredness or low energy for “more than half the days” or “nearly every day” during the preceding two weeks, based on item 4 of the Patient Health Questionnaire-9 (PHQ-9). Low physical activity was determined from weekly energy expenditure estimated from moderate- and vigorous-intensity occupational and leisure-time physical activity and transport-related walking. The KNHANES physical activity questionnaire, which is based on the Global Physical Activity Questionnaire (GPAQ), records the number of days per week and the usual time per day spent in vigorous- and moderate-intensity occupational activity, transport-related walking or cycling, and vigorous- and moderate-intensity leisure-time activity. For each domain, weekly duration was calculated as days per week multiplied by minutes per day. Metabolic equivalent (MET) values of 8.0 for vigorous-intensity activity and 4.0 for moderate-intensity and transport-related activity were assigned according to the GPAQ analysis guide. Weekly energy expenditure (kcal/week) was calculated as 8 × body weight (kg) × vigorous-intensity activity (hours/week) + 4 × body weight (kg) × moderate-intensity and transport-related activity (hours/week). In the original Fried phenotype criteria, low physical activity was defined as the sex-specific lowest 20% of energy expenditure. Because 24.8% of men and 25.2% of women had an estimated energy expenditure of 0 kcal/week, the 20th-percentile cutoff was 0 kcal/week in both sexes. Weakness was defined as low handgrip strength, using sex-specific cutoffs of <28 kg for men and <18 kg for women, consistent with the criteria proposed by the Asian Working Group for Sarcopenia [17]. Finally, slowness was operationalized using self-reported mobility limitation on the EQ-5D mobility item as a proxy for impaired mobility. Participants reporting either “I have some problems in walking about” or “I am confined to bed” were considered positive for the slowness criterion.

### 2.4. Covariates

Covariates were selected based on their established or plausible associations with frailty and nutritional status and their consistent availability in both the 2022 and 2024 KNHANES datasets. These included sociodemographic characteristics, comorbidities, health-related behaviors, and body mass index (BMI). Sociodemographic variables included age, sex, household income (upper two quartiles vs. lower two quartiles), educational level (middle school or lower vs. high school or higher), and living with a spouse (yes vs. no). Hypertension was defined as systolic blood pressure ≥ 140 mmHg, diastolic blood pressure ≥90 mmHg, or current use of antihypertensive medication. Diabetes mellitus was defined as fasting plasma glucose ≥ 126 mg/dL, glycated hemoglobin ≥ 6.5%, a physician diagnosis of diabetes, or use of glucose-lowering medication. Dyslipidemia was defined as total cholesterol ≥ 240 mg/dL or current use of lipid-lowering medication. Chronic kidney disease was defined as an estimated glomerular filtration rate (eGFR), calculated using the Chronic Kidney Disease Epidemiology Collaboration (CKD-EPI) equation, <60 mL/min/1.73 m^2^, or a physician diagnosis of kidney disease. Asthma was defined as a physician diagnosis of asthma. Depression was defined as a total Patient Health Questionnaire-9 (PHQ-9) score ≥10. Anemia was defined as a hemoglobin level < 12 g/dL in women and <13 g/dL in men. Current smoking was defined as having smoked at least 100 cigarettes during the lifetime and currently smoking conventional cigarettes. Current alcohol drinking was defined as alcohol consumption at least once per month during the previous year. BMI was calculated as body weight in kilograms divided by height in meters squared.

### 2.5. Statistical Analysis

All statistical analyses accounted for the complex sampling design of KNHANES, incorporating sampling weights, stratification variables, and primary sampling units. Continuous variables are presented as survey-weighted means with standard errors (SEs), and categorical variables as unweighted counts with survey-weighted percentages. Differences in participant characteristics were evaluated using complex-samples general linear models for continuous variables and Rao–Scott–adjusted chi-square tests for categorical variables. Survey-weighted logistic regression models were used to estimate odds ratios (ORs) and 95% confidence intervals (CIs) for the association between AMDR non-adherence and frailty, with AMDR-adherent participants serving as the reference group. Sequential models were constructed as follows: Model 1 adjusted for age, sex, household income, educational level, and living with a spouse; Model 2 additionally adjusted for hypertension, diabetes mellitus, dyslipidemia, asthma, chronic kidney disease, depression, anemia, current smoking, and alcohol consumption; and Model 3 additionally adjusted for BMI. Analyses were performed separately by energy sufficiency. Associations between adherence to the AMDR for each individual macronutrient and frailty were also examined. To characterize dose–response relationships and evaluate nonlinearity, restricted cubic spline (RCS) models—with knots placed at the 10th, 50th, and 90th percentiles of each macronutrient’s percentage-of-energy distribution—were fitted separately within the energy-sufficient and energy-insufficient strata, using the overall sample median as the reference value. Interaction between each macronutrient (modeled continuously) and energy-sufficiency status was formally tested. Complex-samples analyses were performed using IBM SPSS Statistics version 25.0 (IBM Corp., Armonk, NY, USA); RCS models and corresponding figures were generated using R version 4.6.1 (R Foundation for Statistical Computing, Vienna, Austria). A two-sided *p* < 0.05 was considered statistically significant for all analyses.

## 3. Results

### 3.1. Participant Characteristics

Among participants with available dietary data, those excluded because of incomplete frailty data (*n* = 603) were older and had less favorable socioeconomic and health profiles, lower physical activity and energy intake, and lower adherence to the overall, carbohydrate, and fat AMDRs than those with complete frailty data (*n* = 2843) (Table A2). After further excluding 36 participants with extreme energy intake and 4 with missing BMI data, the final analytic sample comprised 2803 participants. After excluding 36 participants with extreme energy intake and 4 with missing BMI data, the final sample comprised 2803 participants, of whom 1878 were energy-insufficient (<30 kcal/kg/day) and 925 were energy-sufficient (≥30 kcal/kg/day). (Table 1). Compared with the energy-insufficient group, the energy-sufficient group had a higher proportion of men, higher household income and educational attainment, and a higher proportion living with a spouse. The energy-sufficient group also had a lower prevalence of hypertension, dyslipidemia, and chronic kidney disease. Frailty prevalence was lower in the energy-sufficient group (6.1%) than in the energy-insufficient group (9.5%, *p* = 0.006). Overall AMDR adherence did not differ significantly between the energy-sufficient and energy-insufficient groups (29.1% vs. 26.2%, *p* = 0.222). However, adherence to the carbohydrate and protein AMDRs was higher in the energy-sufficient group (41.2% vs. 35.0%, *p* = 0.012; and 86.6% vs. 83.3%, *p* = 0.029, respectively), whereas fat AMDR adherence was similar between the groups (56.2% vs. 54.2%, *p* = 0.433).

### 3.2. Overall AMDR Adherence and Frailty According to Energy Sufficiency

In the total population, non-adherence to overall AMDR was associated with higher odds of frailty in the crude model (OR 1.82, 95% CI 1.27–2.60, *p* = 0.001) and remained associated after adjustment for sociodemographic factors (Model 1: OR 1.63, 95% CI 1.12–2.38, *p* = 0.011) and after additional adjustment for comorbidities and health behaviors (Model 2: OR 1.57, 95% CI 1.06–2.34, *p* = 0.025) (Table 2). After further adjustment for BMI, the association did not reach statistical significance (Model 3: OR 1.50, 95% CI 0.99–2.27, *p* = 0.053). Among energy-sufficient participants, non-adherence to overall AMDR was associated with frailty in the crude model (OR 3.45, 95% CI 1.48–8.05, *p* = 0.004) and remained significant across sequential models, including the fully adjusted model (Model 3: OR 5.58, 95% CI 1.59–19.67, *p* = 0.008). Among energy-insufficient participants, non-adherence to overall AMDR was associated with frailty in the crude model (OR 1.50, 95% CI 1.00–2.24, *p* = 0.048), but this association was attenuated with sequential adjustment and was not significant in the fully adjusted model (Model 3: OR 1.08, 95% CI 0.69–1.68, *p* = 0.739).

### 3.3. Macronutrient-Specific AMDR Adherence and Frailty

In the total population, non-adherence to the fat AMDR was significantly associated with frailty (OR 1.42, 95% CI 1.04–1.95, *p* = 0.027); associations for carbohydrate (OR 1.30, 95% CI 0.90–1.88, *p* = 0.164) and protein (OR 1.40, 95% CI 0.89–2.18, *p* = 0.143) were not significant (Table 3). Among energy-sufficient participants, non-adherence to all three macronutrient-specific AMDRs was associated with higher odds of frailty in a similar direction (carbohydrate: OR 2.15, 95% CI 1.00–4.65; protein: OR 2.63, 95% CI 1.02–6.84; fat: OR 2.38, 95% CI 1.19–4.77). Statistical significance was reached for protein (*p* = 0.046) and fat (*p* = 0.015), whereas the association for carbohydrate was borderline (*p* = 0.051). Among energy-insufficient participants, none of the macronutrient-specific associations were statistically significant (carbohydrate: OR 1.10, 95% CI 0.72–1.68, *p* = 0.672; protein: OR 1.08, 95% CI 0.67–1.77, *p* = 0.746; fat: OR 1.20, 95% CI 0.84–1.71, *p* = 0.317).

### 3.4. Dose–Response Associations Between Macronutrient Distribution and Frailty

**Figure 2:**
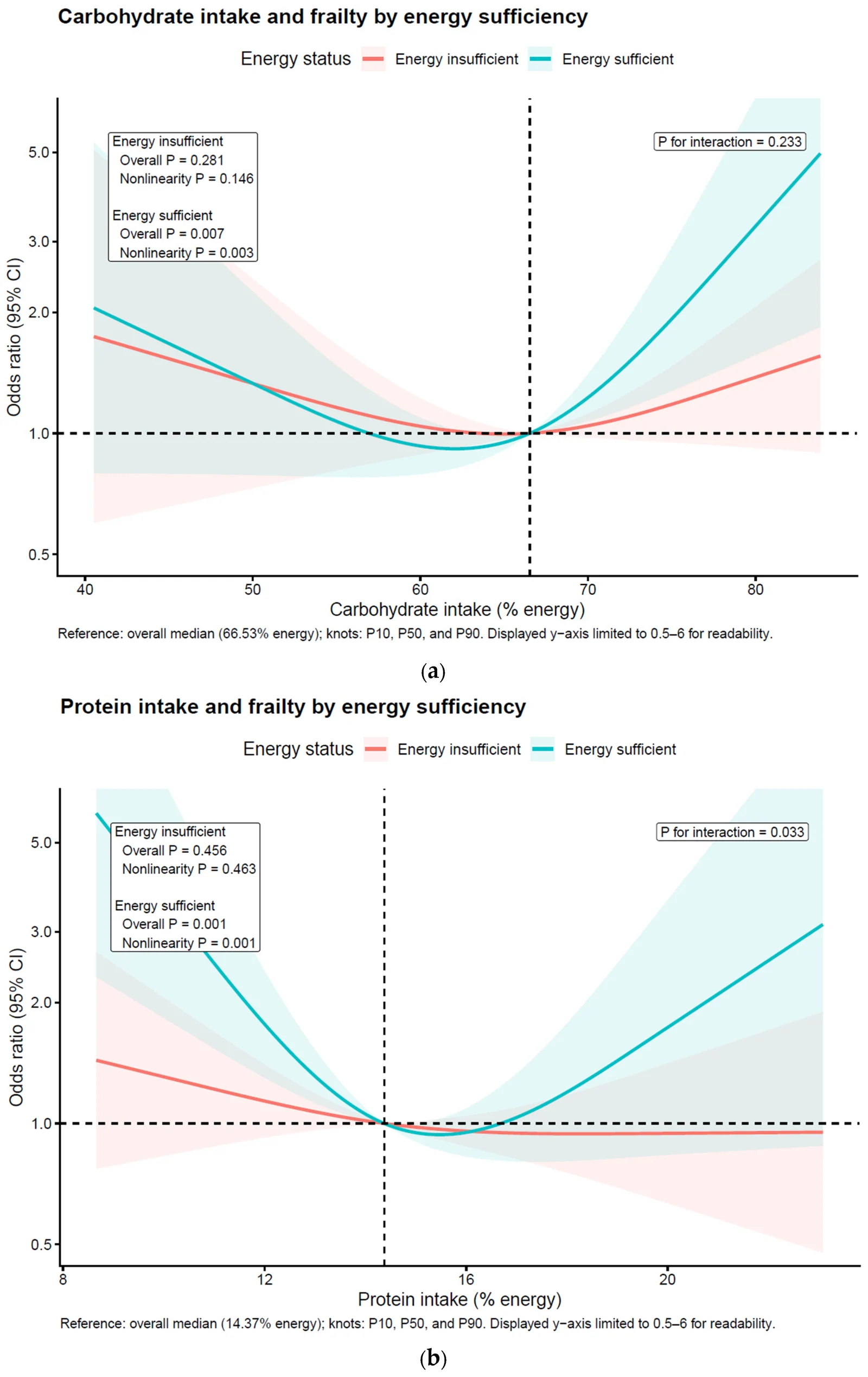

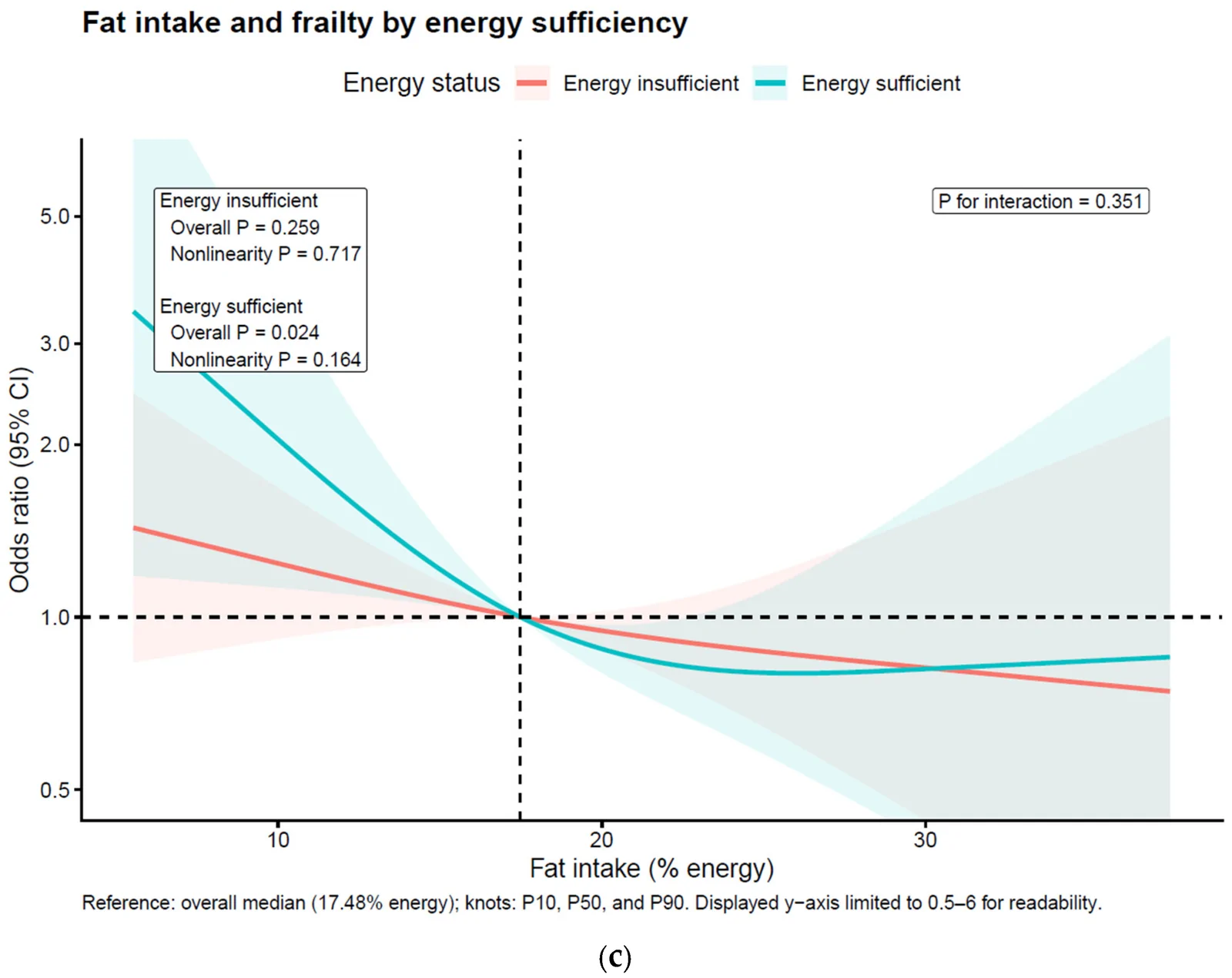
Restricted cubic spline curves show the associations of (**a**) carbohydrate, (**b**) protein, and (**c**) fat intake (% of total energy) with frailty, stratified by energy sufficiency. Solid lines represent odds ratios and shaded areas represent 95% confidence intervals. Knots were placed at the 10th, 50th, and 90th percentiles, with the overall sample median as the reference. Models were adjusted for age, sex, household income, educational level, living with a spouse, hypertension, diabetes mellitus, dyslipidemia, asthma, chronic kidney disease, depression, anemia, current smoking, alcohol consumption, and body mass index. Energy sufficiency was defined as ≥30 kcal/kg/day. *p* values for overall association, nonlinearity, and interaction are shown in each panel.

Restricted cubic spline models were used to examine the dose–response relationship between each macronutrient’s percentage of total energy intake and the odds of frailty, stratified by energy sufficiency (Figure 2). The spline models were adjusted for the same covariates as Model 3. Among energy-sufficient participants, carbohydrate and protein intake showed significant U-shaped associations with the odds of frailty, whereas fat intake showed an inverse association without significant evidence of nonlinearity. None of the three macronutrients showed a significant dose–response association with frailty among energy-insufficient participants. Of the three macronutrients, only protein showed a statistically significant interaction between continuous intake and energy sufficiency (*p* for interaction = 0.033), whereas the interactions for carbohydrate (*p* for interaction = 0.233) and fat (*p* for interaction = 0.351) were not significant.

## 4. Discussion

In this nationally representative study of Korean adults aged ≥65 years, we examined the association between frailty and adherence to the recommended distribution of carbohydrate, protein, and fat with particular consideration of overall energy sufficiency. In the overall population, non-adherence to the overall AMDR was associated with higher odds of frailty in partially adjusted models, although the association was attenuated after additional adjustment for BMI. When stratified by energy sufficiency, overall AMDR non-adherence remained significantly associated with frailty among energy-sufficient participants, whereas no significant association was observed among energy-insufficient participants. In nutrient-specific analyses, non-adherence to carbohydrate, protein, and fat AMDRs showed consistently higher odds of frailty in the energy-sufficient group, reaching statistical significance for protein and fat and borderline significance for carbohydrate. Taken together, AMDR non-adherence was associated with frailty primarily among energy-sufficient older adults, suggesting that the association between macronutrient balance and frailty may differ according to energy adequacy.

As noted above, the AMDR reflects the balance of energy from carbohydrate, protein, and fat rather than absolute nutrient intake. Rather than specifying absolute amounts of individual macronutrients, it defines acceptable ranges for the relative contributions of macronutrients to total energy intake that are compatible with nutritional adequacy and reduced chronic disease risk [12,18]. Thus, intake outside the AMDR reflects an imbalanced macronutrient distribution rather than simply an inadequate or excessive absolute intake of a single nutrient. Accordingly, associations with individual macronutrient AMDR categories should be interpreted as reflecting differences in overall macronutrient distribution rather than independent effects of carbohydrate, protein, or fat in isolation.

A key finding of this study was that non-adherence to the overall AMDR was associated with higher odds of frailty among older adults with sufficient energy intake, whereas no significant association was observed among those with energy insufficiency. However, only 63 participants with frailty were included in the energy-sufficient group, and the wide confidence interval around the estimate indicates limited statistical precision. Therefore, the magnitude of this association should be interpreted cautiously. This finding suggests that adequate energy intake appears to provide an important nutritional context in which the relative balance of macronutrients becomes relevant to frailty. Adequate energy intake is essential for maintaining body weight, muscle mass, and physical function in older adults. Consistent with this, previous epidemiological studies have linked low energy intake to frailty; for example, in the InCHIANTI study, an energy intake of ≤21 kcal/kg/day was independently associated with a higher prevalence of frailty [19]. Similarly, using doubly labeled water–calibrated energy intake to overcome the reporting bias inherent in self-reported dietary assessment, a study of community-dwelling older adults in Japan found a U- or J-shaped relationship between energy intake and frailty prevalence, with the lowest prevalence observed at approximately 40 kcal/kg/day [20]. The ESPEN guideline similarly recommends approximately 30 kcal/kg/day as a general guiding value for energy intake in older adults, based on estimated resting energy expenditure and usual physical activity, while emphasizing the need for individual adjustment according to nutritional and clinical status [16]. Although 30 kcal/kg/day is a practical guiding value rather than a validated cutoff, it was used in the present study as an operational criterion for stratification. Expressing energy intake relative to body weight is also consistent with the approaches used in the InCHIANTI and Japanese studies described above [19,20]. Under energy deficit, amino acids may be diverted toward energy production rather than protein synthesis, impairing muscle protein synthesis even when amino acid intake is adequate [14,21,22]. In older adults with insufficient habitual energy intake, similar catabolic pressures could predominate regardless of how the available energy is distributed among macronutrients, potentially masking any association between macronutrient balance and frailty. This interpretation is partly supported by the findings of Kang et al., who reported inverse associations of total and animal protein intake with frailty only among energy-sufficient Korean older adults, whereas plant protein intake was inversely associated with frailty regardless of energy sufficiency [15]. Similarly, in the Newcastle 85+ Study, the inverse association between protein intake and progression to frailty was attenuated after adjusting for energy intake [23]. These results collectively support the importance of considering energy adequacy when evaluating the association between macronutrient balance and frailty.

Protein provides a biologically plausible link to frailty, since aging-related anabolic resistance increases the importance of adequate protein and amino acid availability for maintaining muscle mass and function [24]. Protein showed a distinct pattern in our study: among energy-sufficient participants, non-adherence to the protein AMDR was associated with higher odds of frailty, the spline analysis showed a pronounced nonlinear association, and protein was the only macronutrient with a significant interaction with energy sufficiency. Previous studies of protein and frailty have been heterogeneous. A meta-analysis by Coelho-Junior et al. (2018) [25] reported a significant inverse association between protein intake and frailty (OR 0.67, 95% CI 0.56–0.82). Another meta-analysis by the same group, pooling 12 cross-sectional and five longitudinal studies, found no significant cross-sectional association regardless of how protein was expressed, but an inverse association in longitudinal analyses [9]. In the Women’s Health Initiative cohort, higher total protein intake expressed as a percentage of energy was associated with a dose-dependent lower risk of incident frailty [26], whereas in the Rotterdam Study, protein intake itself was not independently associated with frailty once an energy-partition model was applied—only total energy intake, not protein specifically, was associated with lower frailty [27]. In the Tromsø Study, higher protein intake expressed as g/kg body weight was inversely associated with pre-frailty/frailty, whereas no association was observed when protein intake was expressed relative to energy intake as g/MJ [10]. Findings have also differed by protein source and by the metric used to express intake: in a Spanish cohort, higher total and animal protein, but not plant protein, was protective [28], whereas among older women in the Nurses’ Health Study the opposite pattern was observed—plant protein was protective and animal protein was not [29]. A recent dose–response meta-analysis of prospective cohorts found that higher consumption of total and plant protein was associated with a reduced risk of frailty [30]. Differences in how protein intake was defined—by source, unit, and adjustment for energy—likely account for much of this heterogeneity. Different analytic approaches may reveal complementary aspects of the protein–frailty association, as illustrated by the Kyoto-Kameoka Study. In this study, higher protein intake was generally associated with lower frailty prevalence, whereas spline analyses revealed a more complex nonlinear pattern—a weak U-shaped association in men and an L-shaped association in women [31]. Similarly, our spline analysis provided complementary information beyond the AMDR-based categorical analysis by demonstrating a nonlinear association between protein energy contribution and frailty among energy-sufficient participants. These findings underscore the value of examining protein intake both in relation to established dietary reference ranges and continuously, particularly when the underlying association may be nonlinear.

Carbohydrate and fat showed different patterns of association with frailty. Among energy-sufficient participants, carbohydrate %E showed a nonlinear association, with higher odds of frailty at both lower and higher intakes. Whereas previous studies have predominantly linked higher carbohydrate intake with greater frailty, our U-shaped pattern suggests that both low and high carbohydrate energy contributions may be unfavorable. Nonetheless, the higher-intake side of this pattern is consistent with previous Korean studies linking carbohydrate-dominant dietary patterns to frailty. In the Korean Frailty and Aging Cohort Study [11], carbohydrate intake exceeding 65% of energy was associated with a higher prevalence of frailty, and isocaloric replacement of fat with carbohydrate was associated with greater frailty, particularly among participants with low energy intake. Using data from the 9th KNHANES (2022–2023), Yu and Chung (2026) found that frail older adults had a higher proportion of energy from carbohydrate and a lower proportion from fat than robust participants [32]. A gender-stratified analysis of the 2009–2020 KNHANES by Kim et al. (2025) likewise showed increasing carbohydrate and decreasing fat %E with worsening frailty among women, with a large majority of frail participants exceeding the carbohydrate AMDR upper bound [13]. These findings may be particularly relevant given the relatively high habitual carbohydrate intake among Korean older adults [12,33]. In contrast, in the Newcastle 85+ Study, robust and frail participants had similar carbohydrate energy contributions (48.0% vs. 49.5%), suggesting that associations with macronutrient distribution may differ across populations with different habitual dietary patterns [23].

The 2025 KDRI also lowered the lower bound of the carbohydrate AMDR from 55% to 50% of total energy, partly reflecting epidemiological evidence of a U-shaped association between carbohydrate energy contribution and mortality, with the lowest mortality risk observed at approximately 50–55% of energy [12,34]. Although mortality and frailty are distinct outcomes, the nonlinear pattern observed in our spline analysis is broadly compatible with the rationale underlying a recommended range rather than a single directional target for carbohydrate intake. Carbohydrate quality may also contribute to the association at higher carbohydrate levels. Diets rich in refined carbohydrates and added sugars may promote adverse glycemic responses and a pro-inflammatory metabolic environment, potentially contributing to muscle catabolism and impaired physical function [35,36]. Consistent with this possibility, higher simple-carbohydrate intake, but not total or complex carbohydrate intake, has been prospectively associated with incident physical frailty [37]. Thus, differences in carbohydrate quality may partly contribute to the observed association, although carbohydrate quality was not assessed in our study.

Fat showed a different pattern: among energy-sufficient participants, higher fat %E was associated with progressively lower odds of frailty. Given the compositional nature of macronutrient intake, however, this finding should be interpreted together with the carbohydrate results rather than as an independent protective association of fat. Because carbohydrate and fat constitute major reciprocal components of total energy intake, lower fat %E may partly represent the counterpart of a carbohydrate-dominant dietary pattern, consistent with the Korean studies described above [11,13,32]. The observed association may also partly reflect underlying food sources. Many foods contributing dietary fat, including meat, fish, dairy products, nuts, and seeds, also provide protein and other nutrients relevant to the maintenance of muscle mass and physical function. Thus, higher fat %E may partly reflect greater consumption of foods that simultaneously contribute protein and other nutrients relevant to frailty, rather than indicating a direct protective effect of fat itself. Because our AMDR-based analysis did not distinguish food sources or fat quality, this possibility could not be examined directly. Taken together, these findings highlight the importance of considering carbohydrate and fat as interdependent components of overall dietary composition rather than interpreting higher fat intake as protective per se.

Importantly, the AMDR reflects the relative distribution of macronutrients rather than their nutritional quality or food sources. Therefore, the observed associations should not be interpreted as isolated effects of carbohydrate, protein, or fat per se. Individuals with similar macronutrient distributions may differ substantially in the types of foods consumed, such as refined versus fiber-rich carbohydrate sources, or animal- versus plant-based sources of protein and fat. Thus, our findings may partly reflect broader dietary patterns and food choices accompanying AMDR adherence or non-adherence rather than the effects of individual macronutrients alone.

This study has several strengths. First, we used data from KNHANES, a nationally representative survey, and accounted for its complex, multistage sampling design, supporting the generalizability of our findings to community-dwelling Korean older adults. Second, we applied the newly revised 2025 Korean Dietary Reference Intakes to define AMDR adherence, providing evidence relevant to current national dietary recommendations. Third, by examining associations according to energy sufficiency, we were able to evaluate macronutrient balance within the broader context of overall nutritional adequacy.

Several limitations should also be considered. First, the cross-sectional design precludes inference regarding temporality or causality, and reverse causation is possible because frailty itself may influence appetite, food preparation capacity, and both the amount and composition of dietary intake. Second, frailty was assessed using a modified Fried phenotype, with some components operationalized using proxy measures available in KNHANES. Although these measures may not fully capture the original Fried criteria, they allowed a standardized assessment of the major frailty domains within the available survey data. Third, dietary intake was assessed using a single 24-h recall, which may not adequately represent usual long-term intake and is susceptible to day-to-day variation, recall error, and underreporting. Such underreporting may have misclassified some participants with adequate habitual intake as energy-insufficient, potentially contributing to the relatively high proportion (67%) in this stratum and affecting the composition and precision of the energy-sufficiency strata and the stratum-specific associations. Fourth, the 30 kcal/kg/day threshold was used as an operational criterion rather than a validated cutoff for individual energy sufficiency, and the results may differ with alternative definitions of energy adequacy. Fifth, the AMDR-based approach captures macronutrient proportions but not differences in nutrient quality or food sources. Sixth, participants excluded because of incomplete frailty data were older and had less favorable socioeconomic, health, and nutritional profiles than those with complete data, suggesting potential selection bias. Because frailty status was unavailable in these participants, the direction and magnitude of this bias cannot be determined. Finally, residual confounding cannot be excluded because several clinically relevant conditions associated with frailty, including stroke, cardiovascular disease, cancer, and osteoporosis, could not be included as covariates because they were not consistently assessed in both survey years.

## 5. Conclusions

Our findings suggest that both adequate total energy intake and balanced macronutrient distribution are relevant to frailty among older adults. The association between AMDR non-adherence and frailty was apparent among individuals with sufficient energy intake, suggesting that the relationship between macronutrient distribution and frailty may differ according to energy sufficiency. These findings support consideration of both energy adequacy and macronutrient distribution in the nutritional assessment of older adults, while prospective studies are needed to establish temporality and further clarify these associations.

## Figures and Tables

**Table 1:** Baseline characteristics according to energy sufficiency.

Characteristic	Energy-Insufficient (*n* = 1878)	Energy-Sufficient (*n* = 925)	*p*
***Sociodemographic characteristics***			
Age (years)	72.7 ± 0.2	72.1 ± 0.2	0.009
Sex (male)	739 (41.1)	495 (53.3)	<0.001
Income (upper two quartiles)	479 (28.4)	314 (36.2)	0.001
Education (≥high school)	608 (34.8)	441 (50.8)	<0.001
Living with spouse	1258 (67.3)	706 (77.7)	<0.001
***Comorbidities***			
Hypertension	1240 (66.1)	475 (51.4)	<0.001
Diabetes mellitus	511 (29.6)	234 (25.9)	0.085
Dyslipidemia	856 (49.0)	381 (43.3)	0.006
Anemia	286 (15.1)	129 (14.5)	0.710
Asthma	87 (5.0)	37 (3.9)	0.238
Chronic kidney disease	217 (11.0)	73 (7.8)	0.023
Depression	56 (2.9)	25 (2.9)	0.984
***Health behaviors***			
Current smoking	154 (8.3)	99 (9.8)	0.241
Current alcohol drinking	601 (32.5)	363 (39.5)	0.003
Exercise (kcal/week)	609.2 ± 30.1	675.7 ± 43.5	0.185
***Dietary intake/AMDR adherence***			
AMDR adherence (all three)	462 (26.2)	262 (29.1)	0.222
Carbohydrate within AMDR	620 (35.0)	367 (41.2)	0.012
Fat within AMDR	974 (54.2)	511 (56.2)	0.433
Protein within AMDR	1558 (83.3)	798 (86.6)	0.029
Energy intake (kcal/day)	1330.0 ± 10.7	2275.7 ± 20.6	<0.001
***Frailty outcome***			
Frailty (Fried, %)	189 (9.5)	63 (6.1)	0.006
***Anthropometry***			
Body mass index (kg/m^2^)	24.7 ± 0.1	22.9 ± 0.1	<0.001

Data are presented as survey-weighted means ± SEs for continuous variables and unweighted counts (survey-weighted percentages) for categorical variables. *p* values were obtained using complex-samples general linear models for continuous variables and Rao–Scott–adjusted chi-square tests for categorical variables. Energy sufficiency was defined as a total energy intake ≥ 30 kcal/kg/day. Abbreviations: AMDR, Acceptable Macronutrient Distribution Range; SE, standard error.

**Table 2:** Association between overall AMDR adherence and frailty according to energy sufficiency.

Population	Crude	*p*	Model 1	*p*	Model 2	*p*	Model 3	*p*	*p* for Interaction ^1^
***Total*** **(*N* = 2803)**									-
AMDR	1 (Reference)	-	1 (Reference)	-	1 (Reference)	-	1 (Reference)	-	
Non-AMDR	1.82 (1.27–2.60)	0.001	1.63 (1.12–2.38)	0.011	1.57 (1.06–2.34)	0.025	1.50 (0.99–2.27)	0.053	
***Energy-sufficient*** **(*n* = 925)**									0.025
AMDR	1 (Reference)	-	1 (Reference)	-	1 (Reference)	-	1 (Reference)	-	
Non-AMDR	3.45 (1.48–8.05)	0.004	4.08 (1.58–10.51)	0.004	5.73 (1.69–19.43)	0.005	5.58 (1.59–19.67)	0.008	
***Energy-insufficient*** **(*n* = 1878)**									
AMDR	1 (Reference)	-	1 (Reference)	-	1 (Reference)	-	1 (Reference)	-	
Non-AMDR	1.50 (1.00–2.24)	0.048	1.27 (0.84–1.93)	0.256	1.15 (0.74–1.77)	0.537	1.08 (0.69–1.68)	0.739	

ORs (95% CIs) for frailty (Fried phenotype, ≥3 criteria) associated with non-adherence to the AMDR were estimated using complex-samples logistic regression, with adherence to all three macronutrient ranges as the reference. Model 1 was adjusted for age, sex, household income, educational level, and living with a spouse; Model 2 was additionally adjusted for hypertension, diabetes mellitus, dyslipidemia, asthma, chronic kidney disease, depression, anemia, current smoking, and alcohol consumption; and Model 3 was additionally adjusted for body mass index. Energy sufficiency was defined as a total energy intake ≥ 30 kcal/kg/day. ^1^ *p* for interaction between AMDR adherence and energy sufficiency was derived from the multiplicative interaction term in the fully adjusted model (Model 3). Abbreviations: AMDR, Acceptable Macronutrient Distribution Range; CI, confidence interval; OR, odds ratio.

**Table 3:** Association between macronutrient-specific AMDR adherence and frailty according to energy sufficiency.

Population	Carbohydrate	*p*	Protein	*p*	Fat	*p*
***Total*** **(*N* = 2803)**						
AMDR	1 (Reference)	-	1 (Reference)	-	1 (Reference)	-
Non-AMDR	1.30 (0.90–1.88)	0.164	1.40 (0.89–2.18)	0.143	1.42 (1.04–1.95)	0.027
***Energy-sufficient*** **(*n* = 925)**						
AMDR	1 (Reference)	-	1 (Reference)	-	1 (Reference)	-
Non-AMDR	2.15 (1.00–4.65)	0.051	2.63 (1.02–6.84)	0.046	2.38 (1.19–4.77)	0.015
***Energy-insufficient*** **(*n* = 1878)**						
AMDR	1 (Reference)	-	1 (Reference)	-	1 (Reference)	-
Non-AMDR	1.10 (0.72–1.68)	0.672	1.08 (0.67–1.77)	0.746	1.20 (0.84–1.71)	0.317

ORs (95% CIs) for frailty (Fried phenotype, ≥3 criteria) associated with non-adherence to each macronutrient-specific AMDR were estimated using complex-samples logistic regression, with adherence to the corresponding AMDR as the reference. Models were adjusted for age, sex, household income, educational level, living with a spouse, hypertension, diabetes mellitus, dyslipidemia, asthma, chronic kidney disease, depression, anemia, current smoking, alcohol consumption, and body mass index. Abbreviations: AMDR, Acceptable Macronutrient Distribution Range; CI, confidence interval; OR, odds ratio. Energy sufficiency was defined as a total energy intake ≥ 30 kcal/kg/day.

## Data Availability

The original data presented in this study are openly available from the KDCA through the KNHANES at https://knhanes.kdca.go.kr/ (accessed on 26 June 2026).

## Abbreviations

The following abbreviations are used in this manuscript:

AMDR	Acceptable Macronutrient Distribution Range
BMI	Body mass index
CKD-EPI	Chronic Kidney Disease Epidemiology Collaboration
eGFR	estimated glomerular filtration rate
EQ-5D	EuroQol 5-Dimension
ESPEN	European Society for Clinical Nutrition and Metabolism
KDCA	Korea Disease Control and Prevention Agency
KNHANES	Korea National Health and Nutrition Examination Survey
PHQ-9	Patient Health Questionnaire-9

## Appendix A

**Table A1:** Comparison of the original Fried frailty phenotype criteria and the modified definitions used in the present study.

Component	Original Fried Phenotype [3]	Present Study	Rationale for Modification
Weight loss	Unintentional weight loss of >10 lb (≈4.5 kg) in the prior year at baseline; ≥5% loss of body weight in the prior year at follow-up	Self-reported weight decrease of ≥3 kg during the preceding year (response categories: 3–6 kg, 6–10 kg, ≥10 kg) among participants who did not report trying to lose weight	The ≥3-kg threshold represents the lowest available category of clinically meaningful weight loss in KNHANES. Restricting the criterion to participants who did not report intentional weight-loss efforts was intended to preserve the original Fried construct of unintentional weight loss.
Exhaustion	Two CES-D items (“I felt that everything I did was an effort”; “I could not get going”); “a moderate amount of the time” (3–4 days) or “most of the time” in the past week	PHQ-9 item 4 (“Feeling tired or having little energy”); response of “more than half the days” or “nearly every day” during the preceding two weeks	The PHQ-9 fatigue item assesses tiredness and low energy, corresponding to the exhaustion construct of the Fried phenotype. A threshold of “more than half the days” or greater was used to identify persistent fatigue rather than occasional symptoms.
Low physical activity	Minnesota Leisure Time Activity Questionnaire (short version); kcal/week in lowest 20% (men < 383 kcal/week; women < 270 kcal/week)	GPAQ-based KNHANES questionnaire (vigorous/moderate occupational, transport, and leisure-time activity); kcal/week = Σ MET × body weight (kg) × weekly hours; sex-specific lowest 20% (cutoff = 0 kcal/week in both sexes)	GPAQ captures physical activity across occupational, transport, and leisure domains and permits energy expenditure to be estimated using MET values. The sex-specific lowest quintile was retained to preserve the relative low-activity approach of the original Fried phenotype; in this population, the 20th-percentile cutoff was 0 kcal/week in both sexes.
Weakness	Grip strength in lowest 20%, stratified by sex and BMI	Handgrip strength < 28 kg (men) or <18 kg (women)	Handgrip strength directly assesses the weakness construct of the Fried phenotype. The AWGS 2019 sex-specific cutoffs (<28 kg in men and <18 kg in women) were used because they provide clinically established thresholds for low muscle strength in Asian older adults.
Slowness	Time to walk 15 ft in slowest 20%, stratified by sex and height	EQ-5D mobility item; self-reported mobility limitation (“some problems in walking about” or “confined to bed”) was considered positive for the slowness criterion.	The EQ-5D mobility item captures perceived limitations in walking and mobility, a functional domain related to the slowness construct of the Fried phenotype. It was used as a proxy for impaired mobility rather than as a direct measure of gait speed.

**Table A2:** Comparison of characteristics between participants included and excluded because of missing frailty data.

Characteristic	Included (*n* = 2843)	Excluded (*n* = 603)	*p*
***Sociodemographic characteristics***			
Age (years)	72.5 ± 0.1	74.5 ± 0.3	<0.001
Sex (male)	1246 (44.9)	233 (37.5)	0.001
Income (upper two quartiles)	805 (31.1)	112 (23.0)	0.005
Education (≥high school)	1059 (39.9)	95 (30.5)	0.040
Living with spouse	1984 (70.5)	360 (59.5)	<0.001
***Comorbidities***			
Hypertension	1739 (61.3)	400 (68.6)	0.002
Diabetes mellitus	755 (28.4)	162 (34.1)	0.032
Dyslipidemia	1249 (47.0)	210 (45.8)	0.679
Anemia	426 (15.2)	126 (21.5)	0.001
Asthma	127 (4.6)	14 (5.2)	0.677
Chronic kidney disease	296 (10.0)	73 (13.1)	0.053
Depression	86 (3.0)	20 (8.0)	0.001
***Health behaviors***			
Current smoking	258 (8.9)	32 (6.1)	0.067
Current alcohol drinking	973 (34.7)	124 (27.4)	0.012
Exercise (kcal/week)	628.1 ± 25.8	429.8 ± 59.1	0.002
***Dietary intake/AMDR adherence***			
AMDR adherence (all three)	730 (27.0)	111 (20.7)	0.007
Carbohydrate within AMDR	995 (36.8)	145 (26.9)	<0.001
Fat within AMDR	1500 (54.6)	268 (46.0)	0.001
Protein within AMDR	2381 (84.1)	502 (82.2)	0.332
Energy intake (kcal/day)	1641.7 ± 14.8	1497.7 ± 29.6	<0.001
***Anthropometry***			
Body mass index (kg/m^2^)	24.1 ± 0.1	23.9 ± 0.2	0.185

## References

[1] Clegg A., Young J., Iliffe S., Rikkert M.O., Rockwood K. (2013). Frailty in elderly people. Lancet.

[2] Kim D.H., Rockwood K. (2024). Frailty in Older Adults. N. Engl. J. Med..

[3] Fried L.P., Tangen C.M., Walston J., Newman A.B., Hirsch C., Gottdiener J., Seeman T., Tracy R., Kop W.J., Burke G. (2001). Frailty in older adults: Evidence for a phenotype. J. Gerontol. A Biol. Sci. Med. Sci..

[4] Dent E., Martin F.C., Bergman H., Woo J., Romero-Ortuno R., Walston J.D. (2019). Management of frailty: Opportunities, challenges, and future directions. Lancet.

[5] Hoogendijk E.O., Afilalo J., Ensrud K.E., Kowal P., Onder G., Fried L.P. (2019). Frailty: Implications for clinical practice and public health. Lancet.

[6] Lorenzo-López L., Maseda A., de Labra C., Regueiro-Folgueira L., Rodríguez-Villamil J.L., Millán-Calenti J.C. (2017). Nutritional determinants of frailty in older adults: A systematic review. BMC Geriatr..

[7] Landi F., Calvani R., Tosato M., Martone A.M., Ortolani E., Savera G., Sisto A., Marzetti E. (2016). Anorexia of Aging: Risk Factors, Consequences, and Potential Treatments. Nutrients.

[8] Rahi B., Colombet Z., Gonzalez-Colaço Harmand M., Dartigues J.F., Boirie Y., Letenneur L., Feart C. (2016). Higher Protein but Not Energy Intake Is Associated with a Lower Prevalence of Frailty Among Community-Dwelling Older Adults in the French Three-City Cohort. J. Am. Med. Dir. Assoc..

[9] Coelho-Junior H.J., Calvani R., Picca A., Tosato M., Landi F., Marzetti E. (2022). Protein Intake and Frailty in Older Adults: A Systematic Review and Meta-Analysis of Observational Studies. Nutrients.

[10] Konglevoll D.M., Hjartåker A., Hopstock L.A., Strand B.H., Thoresen M., Andersen L.F., Carlsen M.H. (2022). Protein Intake and the Risk of Pre-Frailty and Frailty in Norwegian Older Adults. The Tromsø Study 1994–2016. J. Frailty Aging.

[11] Yang N., Lee Y., Kim M.K., Kim K. (2023). Macronutrients intake and physical frailty in Korean older adults: A cohort-based cross-sectional study. Geriatr. Gerontol. Int..

[12] Ministry of Health and Welfare and the Korean Nutrition Society (2025). 2025 Dietary Reference Intakes for Koreans: Summary.

[13] Kim S., Lee H., Kang M. (2025). Frailty and nutritional inadequacy in older Korean adults: A gender-stratified analysis using National Survey Data. PLoS ONE.

[14] Pasiakos S.M., Vislocky L.M., Carbone J.W., Altieri N., Konopelski K., Freake H.C., Anderson J.M., Ferrando A.A., Wolfe R.R., Rodriguez N.R. (2010). Acute energy deprivation affects skeletal muscle protein synthesis and associated intracellular signaling proteins in physically active adults. J. Nutr..

[15] Kang S., Jin Y., Park Y. (2025). Impact of energy intake on the association between protein intake and the prevalence of frailty in older Korean adults: The Korea National Health and Nutrition Examination Survey, 2014–2018. J. Nutr. Health Aging.

[16] Volkert D., Beck A.M., Cederholm T., Cruz-Jentoft A., Hooper L., Kiesswetter E., Maggio M., Raynaud-Simon A., Sieber C., Sobotka L. (2022). ESPEN practical guideline: Clinical nutrition and hydration in geriatrics. Clin. Nutr..

[17] Chen L.-K., Woo J., Assantachai P., Auyeung T.-W., Chou M.-Y., Iijima K., Jang H.C., Kang L., Kim M., Kim S. (2020). Asian Working Group for Sarcopenia: 2019 Consensus Update on Sarcopenia Diagnosis and Treatment. J. Am. Med. Dir. Assoc..

[18] Trumbo P., Schlicker S., Yates A.A., Poos M. (2002). Dietary reference intakes for energy, carbohydrate, fiber, fat, fatty acids, cholesterol, protein and amino acids. J. Am. Diet. Assoc..

[19] Bartali B., Frongillo E.A., Bandinelli S., Lauretani F., Semba R.D., Fried L.P., Ferrucci L. (2006). Low nutrient intake is an essential component of frailty in older persons. J. Gerontol. A Biol. Sci. Med. Sci..

[20] Watanabe D., Yoshida T., Nanri H., Watanabe Y., Date H., Itoi A., Goto C., Ishikawa-Takata K., Sagayama H., Ebine N. (2021). Association Between the Prevalence of Frailty and Doubly Labeled Water-Calibrated Energy Intake Among Community-Dwelling Older Adults. J. Gerontol. A Biol. Sci. Med. Sci..

[21] Gwin J.A., Church D.D., Wolfe R.R., Ferrando A.A., Pasiakos S.M. (2020). Muscle Protein Synthesis and Whole-Body Protein Turnover Responses to Ingesting Essential Amino Acids, Intact Protein, and Protein-Containing Mixed Meals with Considerations for Energy Deficit. Nutrients.

[22] Areta J.L., Burke L.M., Camera D.M., West D.W., Crawshay S., Moore D.R., Stellingwerff T., Phillips S.M., Hawley J.A., Coffey V.G. (2014). Reduced resting skeletal muscle protein synthesis is rescued by resistance exercise and protein ingestion following short-term energy deficit. Am. J. Physiol. Endocrinol. Metab..

[23] Mendonça N., Kingston A., Granic A., Jagger C. (2019). Protein intake and transitions between frailty states and to death in very old adults: The Newcastle 85+ study. Age Ageing.

[24] Bauer J., Biolo G., Cederholm T., Cesari M., Cruz-Jentoft A.J., Morley J.E., Phillips S., Sieber C., Stehle P., Teta D. (2013). Evidence-based recommendations for optimal dietary protein intake in older people: A position paper from the PROT-AGE Study Group. J. Am. Med. Dir. Assoc..

[25] Coelho-Junior H.J., Rodrigues B., Uchida M., Marzetti E. (2018). Low Protein Intake Is Associated with Frailty in Older Adults: A Systematic Review and Meta-Analysis of Observational Studies. Nutrients.

[26] Beasley J.M., LaCroix A.Z., Neuhouser M.L., Huang Y., Tinker L., Woods N., Michael Y., Curb J.D., Prentice R.L. (2010). Protein intake and incident frailty in the Women’s Health Initiative observational study. J. Am. Geriatr. Soc..

[27] Schoufour J.D., Franco O.H., Kiefte-de Jong J.C., Trajanoska K., Stricker B., Brusselle G., Rivadeneira F., Lahousse L., Voortman T. (2019). The association between dietary protein intake, energy intake and physical frailty: Results from the Rotterdam Study. Br. J. Nutr..

[28] Sandoval-Insausti H., Pérez-Tasigchana R.F., López-García E., García-Esquinas E., Rodríguez-Artalejo F., Guallar-Castillón P. (2016). Macronutrients Intake and Incident Frailty in Older Adults: A Prospective Cohort Study. J. Gerontol. A Biol. Sci. Med. Sci..

[29] Struijk E.A., Fung T.T., Rodríguez-Artalejo F., Bischoff-Ferrari H.A., Hu F.B., Willett W.C., Lopez-Garcia E. (2022). Protein intake and risk of frailty among older women in the Nurses’ Health Study. J. Cachexia Sarcopenia Muscle.

[30] Moradi Baniasadi M., Khakbaz M., Azadbakht L. (2026). Dietary intake of total, animal, and plant proteins and risk of frailty: A GRADE-assessed systematic review and dose-response meta-analysis of prospective cohort studies. Clin. Nutr..

[31] Nanri H., Watanabe D., Yoshida T., Yoshimura E., Okabe Y., Ono M., Koizumi T., Kobayashi H., Fujita H., Kimura M. (2022). Adequate Protein Intake on Comprehensive Frailty in Older Adults: Kyoto-Kameoka Study. J. Nutr. Health Aging.

[32] Yu H., Chung S.J. (2026). Association between nutrient intake and frailty status in Korean older adults: A cross-sectional study using the 9th (2022–2023) Korea National Health and Nutrition Examination Survey. Korean J. Community Nutr..

[33] Bae S., Park H. (2025). Age-Stratified Trends in Nutrition and Lifestyle Transitions in Korea: Findings from KNHANES 2013–2022. Nutrients.

[34] Seidelmann S.B., Claggett B., Cheng S., Henglin M., Shah A., Steffen L.M., Folsom A.R., Rimm E.B., Willett W.C., Solomon S.D. (2018). Dietary carbohydrate intake and mortality: A prospective cohort study and meta-analysis. Lancet Public Health.

[35] Laclaustra M., Rodriguez-Artalejo F., Guallar-Castillon P., Banegas J.R., Graciani A., Garcia-Esquinas E., Ordovas J., Lopez-Garcia E. (2018). Prospective association between added sugars and frailty in older adults. Am. J. Clin. Nutr..

[36] Zhu S.M., Chang P., Wang Z., Yang B., Ye H.F. (2025). Association of the dietary inflammation index with frailty in middle-aged and older adults: A systematic review and meta-analysis. Front. Nutr..

[37] Chuy V., Gentreau M., Artero S., Berticat C., Rigalleau V., Pérès K., Helmer C., Samieri C., Féart C. (2022). Simple Carbohydrate Intake and Higher Risk for Physical Frailty Over 15 Years in Community-Dwelling Older Adults. J. Gerontol. A Biol. Sci. Med. Sci..

